# Educational games in geriatric medicine education: a systematic review

**DOI:** 10.1186/1471-2318-10-19

**Published:** 2010-04-23

**Authors:** Ziad Alfarah, Holger J Schünemann, Elie A Akl

**Affiliations:** 1 Geriatrics section, Department of Medicine, Boston University, USA; 2 Department of Clinical Epidemiology and Biostatistics, McMaster University, Canada; 3 Department of Medicine, State University of New York at Buffalo, USA; 4 Department of Family Medicine, State University of New York at Buffalo, USA

## Abstract

**Objective:**

To systematically review the medical literature to assess the effect of geriatric educational games on the satisfaction, knowledge, beliefs, attitudes and behaviors of health care professionals.

**Methods:**

We conducted a systematic review following the Cochrane Collaboration methodology including an electronic search of 10 electronic databases. We included randomized controlled trials (RCT) and controlled clinical trials (CCT) and excluded single arm studies. Population of interests included members (practitioners or students) of the health care professions. Outcomes of interests were participants' satisfaction, knowledge, beliefs, attitude, and behaviors.

**Results:**

We included 8 studies evaluating 5 geriatric role playing games, all conducted in United States. All studies suffered from one or more methodological limitations but the overall quality of evidence was acceptable. None of the studies assessed the effects of the games on beliefs or behaviors. None of the 8 studies reported a statistically significant difference between the 2 groups in terms of change in attitude. One study assessed the impact on knowledge and found non-statistically significant difference between the 2 groups. Two studies found levels of satisfaction among participants to be high. We did not conduct a planned meta-analysis because the included studies either reported no statistical data or reported different summary statistics.

**Conclusion:**

The available evidence does not support the use of role playing interventions in geriatric medical education with the aim of improving the attitudes towards the elderly.

## Background

Educating health care students and professionals in geriatric medicine is important for providing optimal healthcare to the growing elderly population [1]. In fact, attitudes of nursing staff toward the elderly play a critical role in the quality of care provided in long term care facilities [2]. Unfortunately, many health professionals consider geriatric medicine as uninteresting, unrewarding, and even depressing, and prefer to deal with younger patients [3]. Reasons for these negative attitudes include lack of knowledge about the elderly, lack of contact, the age differential between the young health provider and the older patient, as well as certain learning methods that create or reinforce negative stereotypes of the elderly [4,5].

There have been efforts to define competencies required to care for elderly patients. The Association of American Medical Colleges (AAMC) hosted in 2007 a Geriatrics Consensus Conference that devised the Geriatric Competencies for Medical Students [6]. Similarly, the Accreditation Council for Graduate Medical Education (ACGME) has requirements for residency education in Geriatric Medicine in medical specialties [7]. Educational games represent one type of educational interventions that has been used to achieve some of these competencies (e.g. improve knowledge and attitude towards the elderly).

Educational game represents a type of experiential learning where the learner "engages in some activity, looks back at the activity critically, abstracts some useful insight from the analysis and puts the results to work" [2]. Gaming is safe and permits to do what is either impossible or undesirable in real time. It also permits an undesirable action to be stopped and repeated in a more appropriate fashion. In many ways, gaming provides such an environment where no harms can be done and we can learn from our mistakes. A commonly known type of educational games is role playing (also referred to as simulation). Other types of games include social and cooperative games such as board games, card games and games based on television shows.

In board games, the learning process happens indirectly through providing new information, correcting old thoughts or implementing common ideas. Simulation games allow participants to temporarily experience the aging-related changes including memory, senses and functional status as well as some of the stereotypes that older patients encounter. A debriefing session usually follows the game and helps in summarizing the results and in building a more solid knowledge and exposure for future experiences.

Some types of educational games, such as role playing, have been commonly used and studied in the field of geriatric education. Pacala et al reported on their review of 10 years experience of conducting the Aging Game at the University of Minnesota Medical School [8]. They concluded that "Aging Game is an effective tool for stimulating long-lasting awareness and understanding of key issues related to aging and geriatrics". Other studies have found similarly positive results in evaluating the use of role playing games in geriatric education [9-11].

The objective of this study was to systematically review the medical literature to assess the effect of geriatric educational games on the satisfaction, knowledge, beliefs, attitudes and behaviors of health care professionals.

## Methods

### Eligibility criteria

We used the following eligibility criteria:

• Study design: randomized controlled trials (RCT) and controlled parallel-arm clinical trials (CCT). We excluded single arm studies because of the inability to draw inferences about a causal link between any change in the intervention group and the intervention itself in the absence of a control group.

• Participants: members (practitioners or students) of the health care professions.

• Interventions: educational games intended to improve health care of the geriatric population. We defined educational games as "instructional methods requiring the learner to participate in a competitive activity with preset rules" [12]. We excluded studies in which the educational game was not the main component of the intervention.

• Control: Interventions in the control group could have been: a) no intervention; b) standard educational activity; c) untargeted activity. • Outcomes: participants' satisfaction, knowledge, beliefs, attitude, and behaviors.

### Search strategy

The main search strategy consisted of an electronic search in January 2007 of the following databases starting with the dates of their inception: the EPOC Register, the Cochrane Central Register of Controlled Trials (CENTRAL), MEDLINE (1966 onwards), EMBASE (1980 onwards), PsycINFO (1967 onwards), CINAHL (1982 onwards), AMED (1985 onwards), ERIC (1966 onwards), and Dissertation Abstracts Online (1980 onwards). The electronic search strategies used no language restrictions and combined the methodological component of the search strategy of Cochrane Effective Practice and Organization of Care Group (EPOC) with selected MeSH and free text terms relating to educational games (additional file 1). Additional search strategies included searching the Database of Abstracts of Reviews of Effectiveness (DARE) for relevant systematic reviews and screening of the reference list of included studies and relevant reviews. We complemented the search in 2009 by searching the ISI Web of Science for papers citing the studies included in this review.

### Selection and assessment of methodological quality

Two reviewers screened in duplicate and independently the title and abstract of identified citations. The full texts of citations judged potentially eligible by at least one reviewer were also screened in duplicate and independently. The two reviewers resolved their disagreements on eligibility by discussion. We excluded duplicate reports.

We assessed the methodological quality of each included study in a duplicate and independent manner with resolution of disagreements by discussion. The quality criteria were the following: allocation concealment, comparison of baseline characteristics of the 2 study groups, conduct of a baseline test, protection against contamination, use of standardized outcome measurement tools, description of the analytic approach, and percentage of follow-up.

### Data collection

Two reviewers independently extracted the data from each study and resolved their disagreements by discussion or by consulting a third reviewer. For each eligible study, we collected detailed information about the following: study design (and timing of outcome evaluation), the intervention, the control, the participants, the outcomes, the methodological quality and the results. We collected similar information for studies excluded because of their single arm design. In addition, and irrespective of whether a study was eligible or not, we created a database of educational games used in geriatric medicine education.

### Data analysis

We calculated the agreement between the two reviewers for the assessment of eligibility using kappa (κ) statistic. The primary summary statistic we considered in interpreting the effectiveness of the intervention was the difference between the 2 study groups in change of the score from pre to post intervention. We did not conduct a planned meta-analysis because the included studies either reported no statistical data or reported different summary statistics.

## Results

**Figure 1:**
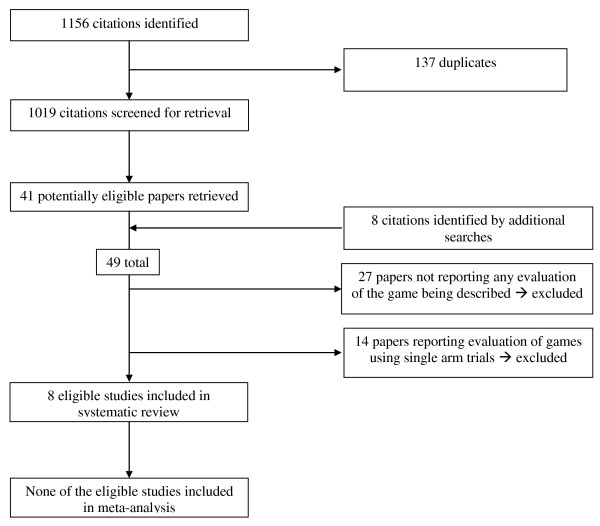
**Study flow**.

Figure 1 shows the information about the literature review and the reasons for inclusion and exclusion of identified citations. The electronic search strategy identified a total number of 1156 citations. We judged 41 of these and 8 additional citations identified through the additional search strategies to be potentially eligible. The full text screening of these 49 citations identified 8 eligible papers. Of the 41 excluded papers, 27 described an eligible game but did not report any evaluation. The remaining 14 excluded papers were ineligible because they used a single arm study design. Agreement between reviewers for eligibility was high (kappa = 0.82). Additional file 2 provides for these 14 single arm studies a detailed description of their design, intervention, participants, outcomes, methodological quality and results. Additional file 3 provides for each game identified through this review (irrespective of whether the corresponding paper was included or not) a detailed description (type, learning objectives, game objective, players, duration, equipment used, and rules) and the list of related publications.

### Overview of included studies

Additional file 4 provides a detailed description of the 8 included studies. All the studies were conducted in United States and published between 1977 and 1995. Participants were nursing staff in long term care institutions (n = 3 studies), healthcare professionals in long term care institutions (n = 1), nursing students (n = 1), medical students (n = 1), occupational therapy students (n = 1), and pharmacy students (n = 1).

In terms of intervention, all eight studies evaluated role playing games: four evaluated the "Into Aging" game [13-16] while each of the other studies examined one of the following four games: "Life Cycle" [3], "Aging Game" [17], "the Geriatric Medication Game" [11], and "Wright's simulation activity" [4]. Despite applying broad inclusion criteria for the control group, the control groups in all studies were unlikely to be efficacious (i.e., bias the results when not applied equally to both groups). One study implemented a lecture-discussion about attitude toward aging [14], another study implemented one hour of role playing during a professional communication class [11], while the remaining 6 studies had no specific intervention.

In terms of outcomes, none of the studies assessed beliefs or behaviors. Two studies assessed learners' satisfaction [3,17]. One of the studies assessed knowledge using a self developed tool [17]. All studies assessed attitude: one study used a self developed tool [11] while the remaining seven studies used one or two of five validated tools. Additional file 5 describes these 5 outcome measurement tools.

### Methodological quality of included studies

Of the 8 included studies, 5 were RCTs and 3 were CCTs [4,11,17]. All but one study [15] conducted a pretest evaluation in addition to the posttest evaluation. Only 2 studies compared the baseline characteristics of the 2 study groups and both reported imbalances [16,17]. As described above, all but one study [11] used validated outcome measure tools. Four studies reported the analytic approach clearly [4,13,16,17]. None of the RCTs reported concealing allocation and none of the studies in general reported efforts to protect against contamination. Four studies reported 100% follow up [3,11,16,17] while the other four did not report on follow-up.

### Quantitative findings

#### Attitude

none of the 4 studies evaluating Into Aging [13-16] or of the respective studies evaluating Life Cycle [3], Wright's simulation activity [4], and Aging Game [17], found a statistically significant difference between the 2 groups in terms of change in attitude from before to immediately after or 3 weeks after the intervention. The study evaluating the Geriatric Medication Game did not report whether the difference between the 2 groups in terms of change in attitude from before to after was statistically significant [11]. The authors reported a statistically significant positive change in attitude from before to after in intervention group, and a higher score for attitude in the intervention group compared with the control group.

#### Knowledge

in the study of Wright's simulation activity, there was no statistically significant or important difference between the 2 groups for knowledge.

#### Satisfaction

both studies evaluating this outcome found high levels of satisfaction among participants.

## Discussion

The systematic review included 8 studies evaluating 5 geriatric role playing games for healthcare professionals. No study evaluating a social and cooperative game was eligible. The included studies suffered from one or more methodological limitations but the overall quality of evidence was acceptable. None of the studies assessed the effects of the games on beliefs or behaviors. None of the 8 studies found a statistically significant difference between the 2 groups in terms of change in attitude. One study assessed the impact on knowledge but found no statistically significant difference between the 2 groups. Two studies found levels of satisfaction among participants to be high.

The major strength of this systematic review is the use of rigorous systematic review methodology including a comprehensive search strategy and duplicate and independent processes for screening, assessing methodological quality of included studies, and abstracting data. Also, we are not aware of any attempt to systematically review the medical literature about the use of educational games in geriatric medicine education.

The findings of this systematic review point to limitations in the literature. All included studies were conducted in the United States, limiting the generalizability of our results to settings with different cultures or other languages. The methodological limitations of the included studies limit the confidence in our conclusions. Our inability to perform a meta-analysis prevented us from uncovering any potential effectiveness of the interventions that individual studies could have been underpowered to detect.

The results of our study suggest that role playing games are not effective interventions for geriatric education. These results are not consistent with the positive conclusions of the review by Pacala et al [8] and of other studies in the field [9-11]. There are two main of reasons for this discrepancy. First, the primary statistic we used to interpret the effectiveness of the intervention was the difference between the 2 study groups in change of the score from pre to post intervention while the authors of the original studies used the change of the score from pre to post in the intervention group. In many of these studies the former statistic (i.e., the review's primary statistic) was not statistically significant while the latter (i.e., statistic reported by the authors) was [4,14]. Second, many of these positive studies were single arm studies which we excluded from our review because of the greater potential for bias.

While the findings of RCTs and CCTs included in this study were consistent, those of single arm studies evaluating role play were not (Additional file 2). In fact, the results for single arm studies reporting both pretest and posttest results for impact on attitude were: improvement in two studies [10,18], no change in one study [2], worsening in one study [9], and unclear impact in one study [19]. In terms of impact on knowledge, one study found improvement [20], while another found no improvement [2]. These discrepancies highlight the importance of the control groups in trials. This is particularly relevant to educational interventions because educational outcomes are often measured pre and post intervention and investigators are tempted to conduct single arm trial and simply measure change of the score from pre to post intervention.

As our findings suggest that role playing games are not effective interventions to improve the attitudes of health care professionals and students toward the elderly, medical educators should consider alternative interventions to attain that goal. One example is the mentors-on-aging program which offers students direct interaction with real-life healthy, active older adults with the aim of instilling positive attitudes toward older people[21]. Another example is the intergenerational service-learning projects in which students are exposed to issues of aging, mental health, nutrition, and fitness through service to another older adults [22].

## Conclusions

The main implication of the review findings for educational practice is that the available evidence does not support the use of role playing interventions in geriatric education with the aim of improving the attitudes towards the elderly. In fact, all of the 8 studies addressing this specific question showed no statistically significant benefit. In addition these interventions require significant effort and time investment on the part of the educator and significant personnel resources [8].

The findings have a number of implications for research in this field. Any future trial of a role playing game in geriatrics should be powered sufficiently to rule out with confidence or uncover any potential effectiveness that the included studies could have been underpowered to detect. On a more general level, medical education trialists need to design their trials as RCTs with high methodological standards [23]), assess relevant educational outcomes such as behavioral change, conduct pre and post intervention outcome assessment, evaluate immediate as well as sustained benefit, and use validated outcome measures. Researchers also need to better report their studies in terms of describing the intervention (to allow replication), the study methodological features (to allow judgement of validity), and the numerical and statistical results (to allow meta-analyzing the results across studies).

## Competing interests

The authors declare that they have no competing interests.

## Authors' contributions

Conception and design: EAA, HJS; screening: ZA, EAA, data abstraction: ZA, EAA, data interpretation: ZA, EAA, HJS; manuscript drafting: ZA, EAA. All authors critically reviewed the content of the report and approved its final version.

## Pre-publication history

The pre-publication history for this paper can be accessed here:

http://www.biomedcentral.com/1471-2318/10/19/prepub

## Supplementary Material

Additional file 1 **Search strategies of electronic databases**. Search strategies of electronic databases. Click here for file

Additional file 2 Characteristics of single arm trials excluded from the systematic review. Click here for file

Additional file 3 Detailed description and list of publications related to game identified through this review. Click here for file

Additional file 4 **Characteristics of randomized controlled trials included in the systematic review**. Characteristics of randomized controlled trials included in the systematic review. Click here for file

Additional file 5 **Validated tools for assessing attitude outcome**. Validated tools for assessing attitude outcome. Click here for file
