# Supplementary material for: Educational games in geriatric medicine education: a systematic review
Source: BMC Geriatr. 2010 Apr 23;10:19. doi: 10.1186/1471-2318-10-19 (PMC2867807; doi:10.1186/1471-2318-10-19)
Supplement: Additional file 1 — Search strategies of electronic databases. Search strategies of electronic databases. [file 1471-2318-10-19-S1.DOC]

**Search strategies of electronic databases**

MEDLINE

1. Video Games/

2. "Play and Playthings"/

3. Games, Experimental/

4. (game? or gaming).tw.

5. structured experience?.tw.

6. or/1-5

7. exp *education,continuing/

8. exp Education, Professional/

9. professional development.tw.

10. exp Learning/

11. ((medical or clinical or professional or clinician) adj (train$ or learn$)).tw.

12. (behavio?r$ adj2 intervention?).tw.

13. or/7-12

14. exp Students, Health Occupations/

15. exp Health Personnel/

16. (provider? or practitioner? or doctor? or gp? or physician? or nurs$).tw.

17. ((health or healthcare or health care or medical) adj (student? or staff or worker? or professional? or personnel)).tw.

18. or/14-17

19. 6 and 13 and 18

EMBASE

1. exp Recreation/

2. Play/

3. Game/

4. (game? or gaming).tw.

5. structured experience?.tw.

6. or/1-5

7. exp Medical Education/

8. exp Paramedical Education/

9. Continuing Education

10. professional development.tw.

11. exp Learning/

12. ((medical or clinical or professional or clinician) adj (train$ or learn$)).tw.

13. (behavio?r$ adj2 intervention?).tw.

14. or/7-13

15. exp Health Personnel/

16. (provider? or practitioner? or doctor? or gp? or physician? or nurs$).tw.

17. ((health or healthcare or health care or medical) adj (student? or staff or worker? or professional? or personnel)).tw.

18. or/15-17

19. 6 and 14 and 18

CINAHL

1. Video Games/

2. "Play and Playthings"/

3. Games/

4. (game? or gaming).tw.

5. structured experience?.tw.

6. or/1-5

7. exp Education, Health Sciences/

8. professional development.tw.

9. exp Learning/

10. exp Teaching Methods/

11. ((medical or clinical or professional or clinician or practitioner or nurs$) adj (train$ or learn$)).tw.

12. (behavio?r$ adj2 intervention?).tw.

13. or/7-12

14. exp Health Personnel/

15. (provider? or practitioner? or doctor? or gp? or physician? or nurs$).tw.

16. ((health or healthcare or health care or medical) adj (student? or staff or worker? or professional? or personnel)).tw.

17. or/14-16

18. 6 and 13 and 17

PsycINFO

1. exp Games/

2. Game Theory/

3. (game? or gaming).tw.

4. structured experience?.tw.

5. or/1-4

6. exp Medical Education/

7. exp Continuing Education/

8. Professional Development/

9. exp Learning/

10. ((medical or clinical or professional or clinician) adj (train$ or learn$)).tw.

11. (behavio?r$ adj2 intervention?).tw.

12. or/6-11

13. exp Health Personnel/

14. (provider? or practitioner? or doctor? or gp? or physician? or nurs$).tw.

15. ((health or healthcare or health care or medical) adj (student? or staff or worker? or professional? or personnel)).tw.

16. or/13-15

17. 5 and 12 and 16

AMED

1. "Play and playthings"/

2. video game?.tw.

3. (game? or gaming).tw.

4. structured experience?.tw.

5. or/1-4

6. exp Education Professional/

7. professional development.tw.

8. exp Learning/

9. ((medical or clinical or professional or clinician) adj (train$ or learn$)).tw.

10. (behavio?r$ adj2 intervention?).tw.

11. or/6-10

12. exp Health Personnel/

13. (provider? or practitioner? or doctor? or gp? or physician? or nurs$).tw.

14. ((health or healthcare or health care or medical) adj (student? or staff or worker? or professional? or personnel)).tw.

15. or/12-14

16. 5 and 11 and 15

ERIC (EBSCOhost)

S1. SU play

S2. SU video games

S3. SU games

S4. TI (game* or gaming)

S5. TI (structured experience*)

S6. S1 or S2 or S3 or S4 or S5

S7. SU medical education

S8. SU continuing education

S9. SU professional development

S10. SU learning

S11. SU medical schools

S12. TI (medical N1 train* or medical N1 learn* or clinical N1 train* or clinical N1 learn* or professional N1 train* or professional N1 learn* or clinician N1 train* or clinician N1 learn*)

S13. TI (behaviour* N2 intervention or behavior* N2 intervention)

S14. S7 or S8 or S9 or S10 or S11 or S12 or S13

S15. SU health personnel

S16. SU nurses

S17. SU physicians

S18. SU medical students

S19. TI (provider* or practitioner* or doctor or doctors or gp* or physician* or nurse* )

S20. TI (health N1 student or health N1 staff or health N1 worker* or health N1 professional* or health N1 personnel or healthcare N1 student or healthcare N1 staff or healthcare N1 worker* or healthcare N1 professional* or healthcare N1 personnel or health care N1 student or health care N1 staff or health care N1 worker* or health care N1 professional* or health care N1 personnel or medical N1 student or medical N1 staff or medical N1 worker* or medical N1 professional* or medical N1 personnel)

S21. S15 or S16 or S17 or S18 or S19 or S20

S22. S6 and S14 and S21

ProQuest Dissertations & Theses: Full Text

(game* or gaming or video game*) OR IF(play*) OR TITLE(structured experience*) AND (medical educat* or medical school*) OR (continuing education) OR (professional development) OR IF (learn*) OR IF (teaching) OR TITLE((medical or clinical or professional or clinician) w/1 (train* or learn*)) OR TITLE((behavior* or behaviour*) w/2 intervention*) AND (provider* or practitioner* or doctor* or gp* or physician* or nurs*) OR IF (health personnel) OR IF(medical student*) OR TITLE((health or healthcare or health care or medical) w/1 (student* or staff worker* or professional* or personnel))
