# Supplementary material for: Educational games in geriatric medicine education: a systematic review
Source: BMC Geriatr. 2010 Apr 23;10:19. doi: 10.1186/1471-2318-10-19 (PMC2867807; doi:10.1186/1471-2318-10-19)
Supplement: Additional file 2 — Characteristics of single arm trials excluded from the systematic review. [file 1471-2318-10-19-S2.DOC]

**Characteristics of single arm trials excluded from the systematic review**

| **Study** | **Study design** | **Intervention** | **Participants** | **Outcomes** | **Methodological quality** | **Results** |
| --- | --- | --- | --- | --- | --- | --- |
| Hoffman 1985 [1] | - Single arm study - Posttest evaluation (immediately post intervention) | - *“Geriatrix”* - Intervention: playing the game in the first session of a required 2-week geriatrics clerkship | - 45 third year medical students on a geriatrics clerkship - Country: USA | - Attitudes - Satisfaction with and evaluation of the game | - Baseline test: no - Standardized outcome measurement tool: no - Analytic approach described: no - Follow-up: 100% | - Ratings were overall positive for attitude, satisfaction and evaluation of the game |
| Crooks 1987[2] | - Single arm study (2 study group analyzed together) - Posttest evaluation (immediately) | - *“Geriatric Challenge Bowl”* - Intervention: 8 teams competing in 4 preliminary, 2 semifinal and one final session over 6 months & during noon conference or grand rounds. - Control: watching the game (as audience) | - 53 residents at a Veterans Affairs medical center (intervention: 21; control: 32) - Country: USA | Form I (both groups)   - Knowledge - Clinical applicability - Attitude change (self development questionnaire) - Educational effectiveness (personal estimation on a 1-5 scale) - Motivation for attendance   Form II (intervention group only)   - Educational experience | - Comparison of baseline characteristics: no - Baseline test: no - Protection against contamination: no - Standardized outcome measurement tool: no - Analytic approach described: yes but not in detail - Follow-up: 52 residents completed form I; 21 residents completed form II | - Evaluations were overall positive and 45% reported high to very high degree of positive attitude change - A moderate correlation between the reported attitude toward geriatrics and the application of learning to the care of geriatric patients. - Residents who felt increase knowledge about the clinical management of geriatric patients spent more time preparing for the game (p<0.05) |
| McVey 1989 [3] | - Single arm study - Posttest evaluation (immediately post intervention) | - *“Aging Game”* - Intervention: playing the game as part of a required introduction to clinical medicine course | - 112 first and second year medical students - Country: USA | - Self reported awareness and sensitivity to the problems of aging | - Follow up: 100% - Baseline test: no - Standardized measurement tool: no - Analytic approach described: no | - More participants agreed than disagreed regarding: enjoyment, learning facts about elderly, learning about own attitude towards elderly, learning how much have to be learnt about geriatric medicine - More participants disagreed than agreed regarding: attitude change, game being waste of time, being convinced of interest in geriatric medicine |
| Galanos 1993 [4] | - Single arm study - Posttest evaluation (1-3 years post intervention) | - *“Aging game”* - Intervention: participation in the game as part of the curriculum (during first or second year) | - 82 medical students (23 second year, 28 third year,31 fourth year) - Country: USA | - Attitudes (i.e., remembering game when encountering elderly, influence on approach to elderly, degree of interest and fun during working with elderly, likeness of having geriatric patients in practice) | - Follow up: 82/300 (27%) - Baseline test: no - Standardized measurement tool: no - Analytic approach described: not clearly | - Authors do not report statistically analyzable data - Histograms suggest no major trends in favor or disfavor of the game - Authors report that there were no differences between 3 medical school classes included in the study |
| Varkey 2006 [5] | - Single arm study - Pretest and posttest (immediately post) | - *“Aging game”* - Intervention: 3 hours session of role playing | - 84 first and second year medical students - Country: USA | - Attitudes and empathy toward the elderly: 11 question modified Maxwell Sullivan Attitude Scale (M&S) [6] - General Attitude towards the elderly: Aging Semantic Differential scale (ASD) [7] | - Follow up: 100% for the posttest - Baseline test: yes. - Standardized measurement tool: yes - Analytic approach described: yes | Attitude and Empathy   - M&S: statistically significant change in 6 of the 8 attitudes questions and a statistically significant increase in empathy based on 3 of 3 questions   General attitude:   - ASD: statistically significant change on 23 out of 32 items |
| Henry 2007 [8] | - Single arm study - Pretest and posttest evaluation (one week before and one week after the test) | - “Aging Game” - Intervention: playing the game during class time | - 156 students in nutrition and dietetics, physical therapy, and long-term care administration - Country: USA | - Attitude towards the elderly: Aging Semantic Differential scale (ASD) [7] - Anxiety about getting older: Anxiety about Aging Scale (AAS). | - Follow up: 145/156 (93%) completed all questions. - baseline test: yes - Standardized outcome measurement tool: yes - Analytic approach described: yes | Attitude:  Worsened: pre=78.84 (SD 18.2), post=82.61 (20.7) (p<0.05)  Anxiety:   - overall worsened pre=55.73(6.00), post=54.21(6.47); difference=-1.52 (p<0.05) - improved for physical appearance, worsened for psychosocial concern and fear of losses, and stable for fear of old people |
| Israel 1992 [9] | - Single arm study - Posttest evaluation (immediately post) | - *“Gerontopoly”* - Intervention: playing the game as part of a geriatric dentistry course | - Phase I: 186 students from different fields - Phase II: 71 first year dental students - Country: USA | Phase I: 22-item questionnaire (5-point Likert-like scales) measuring:   - Reactions to the game - Acceptability of the game   Phase II:   - Knowledge: Palmore’s Facts on Aging Quiz 2 [10] - Change in attitude and empathy towards elderly - Change of awareness of the elderly concern | - Follow up: 100% answered completed post test; no long term follow up - Baseline test: no - Standardized Outcome measurement tool: yes for knowledge - Analytic approach described: partially | - Ratings were overall positive for phase I and phase II trial. - There were no statistically significant differences between the 2 groups for improvement in Palmore quiz scores |
| Cipriani 1995 [11] | - Single arm study - Pretest and Posttest evaluation (immediately post intervention) | - *“Resident Assessment Instrument’ (RAI)* - Intervention: teaching of the RAI during residential courses for health professionals working with nursing homes | - 33 certified nurses and 5 physiotherapists attending one of 2 courses - Country: Italy | - Knowledge: 120 item multiple choice quiz - Knowledge: adapted version of Palmore’s Facts on Aging Quiz 2 [10] - Ability in assessment and care planning and attitudes toward working with the elderly: RAI - Satisfaction with and evaluation of the game | - Follow up: all participants. No long term follow up - Baseline test: yes - Standardized Outcome measurement tool: yes for ability of care planning and attitudes only. - Analytic approach described: not clearly | Knowledge:   - multiple choice quiz: reported as a histogram; overall positive - adapted Palmore test: pre=14.51 (SD=2.06), post=19.29 (SD=2.01); p<0.005 - RAI results: reported as a histogram; overall positive - Results from the satisfactory questionnaire are overall positive |
| Khazadian Figuerosa 1997 [12] | - Single arm study - Posttest evaluation (immediately post, 2 weeks post and 6 months post intervention) | - *“Into aging”* - Intervention: nursing staff development program sessions (total of 12 sessions) | - 149 certified nurse assistants and head nurses - Country: USA | - Self reported change in behavior and attitude | - Follow-up: 107/149 (72%) attended the post class evaluation. 101/149 (69%) returned the post-two week’s questionnaire - Baseline test: no - Standardized measurement tool: no - Analytic approach described: no | - Rating was overall positive. |
| Thomson 1998 [13] | - Single arm study - Pretest and posttest evaluation (4-6 weeks post intervention) | - *“Into Aging”* - Intervention: theoretical and experiential training including *Into Aging* | - 70 nursing assistants in a health care center (of 99 potential participants) - Country: Canada | - Knowledge on aging: Palmore’s Facts on Aging Quiz [10] - Attitude toward the elderly: Kogan Attitude toward Old People scale [14] | - 34 of the 70 participants who completed the pretest completed the posttest - Baseline test: yes - Outcome measures validated: yes - Analytic approach described: yes | Knowledge:   - pre=14.2 (2.7) vs. post=15.1 (3.2); not statistically significant   Attitude:   - pre=135.6 (19.5) vs. post 145.5=(18.5); not statistically significant |
| Lorraine 1998 [15] | - Single arm study - Pretest and posttest evaluation (immediately post intervention) | - *“Aging simulation”* - Intervention: playing the game in a retail store for 3 hours during mandatory geriatric clerkship | - 100 fourth year medical student - Country: USA | - Attitude towards the elderly: Aging Semantic Differential scale (ASD) [7] - Rating of the simulation exercise | - Follow up: 100 % completed the course evaluation. No long term follow-up. - Baseline test: yes - Standardized outcome measure tool : yes - Analytic approach described: yes | Attitude:   - significant improvement from pre to post: 171.59 vs. 148.69 (p<0.001) - Rating of the game was overall positive. |
| Skinner 2000 [16] | - Single arm study - Pretest and posttest evaluation (immediately post intervention) | - *“Sexual dysfunction Trivia Game”* - Intervention: playing the board game | - Staff nurses (participants’ number not reported) - Country: USA | - Knowledge related to physical exam, laboratory tests and treatment options | - Follow up: not reported - Baseline test: yes - Standardized outcome measurement tool: no - Analytic approach described: no | - Answers were overall positive |
| Robinson 2001 [17] | - Single arm study - Pretest and posttest evaluation (immediately post intervention) | - *“Half Full Aging Simulation experience”* - Intervention: simulation game following a short presentation by a geriatrician | - 49 third year medical students - Country: USA | - Attitude towards the elderly: Aging Semantic Differential scale (ASD) [7] | - Follow up: 100%. No long term f/u - Baseline test: yes - Standardized measurement tool: yes - Analytic approach described: not clearly | Attitudes:   - pre= 124.35 vs. post=116.14 (no SD reported; no report whether difference statistically significant for scale) - Changes statistically significant in only one subscale (instrumental-ineffective) (p<0.003) |
| Evans 2005 [18] | - Single arm study - Pretest and posttest evaluation | *“The Geriatric Medication Game”*   - Intervention: role playing during professional communication class | - 102 pharmacy students - Country: USA | - Perception of elderly persons questionnaire (12 items) - Evaluation of the game | - Follow up: 96/102 completed the pre/post test. - Baseline test: yes. - Standardized measurement tool: no - Analytic approach described: no | - Perceptions improved significantly for 8/12 items - Evaluation overall positive |

**References**

1. Hoffman SB, Brand FR, Beatty PG, Hamill LA: **Geriatrix: A role-playing game.** *Gerontologist* 1985, **25:**568-572.

2. Crooks V, Yoshikawa TT: **Geriatric Challenge Bowl: an innovative teaching approach.** *Gerontology & Geriatrics Education* 1987, **7:**67-79.

3. McVey LJ, Davis DE, Cohen HJ: **The 'aging game'. An approach to education in geriatrics.** *JAMA* 1989, **262:**1507-1509.

4. Galanos AN, Cohen HJ, Jackson TW: **Medical education in geriatrics: The lasting impact of the aging game.** *Educational Gerontology* 1993, **19:**675-682.

5. Varkey P, Chutka DS, Lesnick TG: **The Aging Game: improving medical students' attitudes toward caring for the elderly.** *Journal of the American Medical Directors Association* 2006, **7:**224-229.

6. Maxwell AJ, Sullivan N: **Attitudes toward the geriatric patient among family practice residents.** *J Am Geriatr Soc* 1980, **28:**341-345.

7. Rosencranz HA, McNevin TE: **Aging semantic differential.** In *Research Instruments in Social Gerontology.* Edited by Mangen DJ, Peterson WA. Minneapolis: University of Minnesota; 1982

8. Henry BW: **Effects of participation in an aging game simulation activity on the attitudes of Allied Health students toward older adults.** *The internet journal of allied health sciences and practice* 2007, **5**.

9. Israel MD, Dolan TA, Caranasos GJ: **Gerontopoly: Development and testing of a new game in geriatric education.** *Gerontology & Geriatrics Education* 1992, **12:**17-30.

10. Palmore E, Palmore E: **The facts on aging quiz: a review of findings.** *Gerontologist* 1980, **20:**669-672.

11. Cipriani L, Landi F, Sgadari A, Zuccala G, Bernabei R: **A GERONTOLOGICAL CONTINUING-EDUCATION PROGRAM - THE RESIDENT ASSESSMENT INSTRUMENT AS A TEACHING TOOL FOR NURSING-HOME PROFESSIONALS.** *Educational Gerontology* 1995, **21:**683-699.

12. Khazadian-Figueroa MR, Johnson E: **Simulation game: a tool for staff development and its effects on staff behavioral outcomes.** *Journal of Nursing Staff Development* 1997, **13:**223-226.

13. Thomson M: **A Nursing Assistant Training Program in a Long Term Care Setting.** *Gerontology & Geriatrics Education* 1998, **19:**23 - 35.

14. Kogan N, Kogan N: **Attitudes toward old people: the development of a scale and an examination of correlates.** *Journal of Abnormal & Social Psychology* 1961, **62:**44-54.

15. Lorraine V, Allen S, Lockett A, Rutledge CM: **Sensitizing students to functional limitations in the elderly: an aging simulation.** *Family Medicine* 1998, **30:**15-18.

16. Skinner KD: **Creating a game for sexuality and aging: the Sexual Dysfunction Trivia game.** *Journal of Continuing Education in Nursing* 2000, **31:**185-189.

17. Robinson SB, Rosher RB: **Effect of the "half-full aging simulation experience" on medical students' attitudes.** *Gerontology & Geriatrics Education* 2001, **21:**3-12.

18. Evans S, Lombardo M, Belgeri M, Fontane P: **The Geriatric Medication Game in pharmacy education.** *American Journal of Pharmaceutical Education* 2005, **69**.
