# Supplementary material for: Educational games in geriatric medicine education: a systematic review
Source: BMC Geriatr. 2010 Apr 23;10:19. doi: 10.1186/1471-2318-10-19 (PMC2867807; doi:10.1186/1471-2318-10-19)
Supplement: Additional file 3 — Detailed description and list of publications related to game identified through this review. [file 1471-2318-10-19-S3.DOC]

**D**etailed description and list of publications related to game identified through this review

| **Game, related publications** | **Description of the game** | **Rules of the game** |
| --- | --- | --- |
| **Life Cycle**  Chaisson 1977 [1] | - Game type: board game with role playing - Learning objective: to improve the attitude toward the old person; to live different social and medical experiences related to elderly and increase the knowledge about them - Game objective: to chance upon encounters with other individuals as one would in real life - Players: health care personnel in geriatrics. 5-10 players divided into 2 teams: senior citizens and significant others - Duration: 10 hours divided into 3 sessions - Equipment used: poker chips to register the feedback the players describe, or feel wheels in order to select the appropriate descriptors- if they have difficulty labeling the feelings | - The player throws the dice and moves his/her token along the role labeled path of the circular board. The significant others stand by to play the roles of protagonists in difficult situational encounters - A game manager provides cards describing each player part and perspective in a situation to be acted out - When the player lands on a labeled space, he/she has four choices: (1) go ahead and play out a role with a significant other; (2), not play out the role by withdrawing into the retreat circle: (3) take a risk and opt for a more difficult role and advance into inner circle; (4) remove himself from the game entirely by moving into outer rim or death circle - Game manager times and audiotapes or videotapes interactions them for further discussions during the debriefing session and post-game analysis |
| **Into Aging**  Hoffman 1978 [2]  Related:   - Bonstelle 1984 [3] - Nolan 1985 [4] - Hoyt 1987 [5] - Wirth 1987 ([6] - Marte 1988 [7] - Jadlowski 1991 [8] - Hahn-Marsh 1992 [9] - Seibert 1992 ([10] - LeBlanc 1995 [11] - Khazadian-Figuerosa 1997 [12] - Thomson 1998 [13] | - Game type: role playing - Learning objective: to increase staff awareness; to promote positive behaviors and attitudes during interaction with elderly - Game objective: to keep savings, remain independent and retain role identity despite the odds against the player - Players: nursing staff - Duration 90 minutes: 60 minutes for role playing 30 minutes for debriefing - Material needed include wheelchairs, disposable briefs, washcloths, towels, eyeglasses, tissues, pureed food, cutlery, bibs, and safety devices | - Players choose an identity by selecting a name, an age over 65, an occupation, a place of residence and 3 favorite possessions. They are given an allotment of money as their savings and self esteem symbols - Players interact with an instructor at each table. They roll dices and draw cards that represent various life events that older persons might encounter. They also use sensory loss props such as ear plugs and taped eyeglasses - Players experience loss of possessions, money, and self esteem as they progress through the tables. They progress through 3 identity tables: table 1 as an independent lifestyle; table 2 as a semi-independent lifestyle (adult care) and table 3 as a dependent lifestyle (nursing home) - At conclusion of role playing, a debriefing session allows players to express feelings, identify themes and issue in the care of the elderly, and explore stereotypes |
| **The Aging Game**  Robertson 1981 [14]  Related:   - Lye 1983 (modified version) [15] | - Game type: card game - Learning objectives: to communicate factual knowledge; to encourage the development of appropriate attitudes; to foster debate, discussion and argument; to promote peer learning - Game objective: to secure a hand in which all the statements on the cards are true (see rules) - Players: physicians, medical students, nurses, therapists, social workers, and mixed groups of health professionals - Note: the instructor can modify the rules of the game depending on size of group, time available, and prior knowledge of demography or longevity | - The game is played with special cards bearing statements about old age, some of which are true and the other is false - The instructor shuffles and divides the cards between players equally. Every player discards only one card that bears a statement he/she believes to be negative and attempts to assemble a group in which all statements are true - Players subsequently form pairs and partnership with other pairs to obtain the maximum number of correct cards - Time to discuss the relative merits of the cards and to exchange with other groups is allowed every time two groups form a partnership - At the end, the instructor collects the scores and holds discussions about the true discarded cards |
| **The Road of Life**  Menks 1983 [16] | - Game type: board Game - Learning objective: to increase the participants understanding and awareness of some of the common experiences of old age - Game objective: to retain and attain life units as one moves along the road - Players: children in a 5th grade classroom, adolescents, occupational therapy and clinical psychology students - Duration: 25 min to play without discussion - Equipment used: e.g. adapted glasses to simulate tunnel vision | - Each player receives 10 life units’ cards in the beginning and they can choose to invest some of them in purchasing coping cards that modify gains and losses of life units - Players roll the dice, move markers accordingly and follow directions written on the segments of the road of life that they land upon - Players may lose coping cards (e.g. lifelong good health practices, a friend) if they land on physical card segment - They may acquire a disability if they land on “handicap card” segment - The game is over when all of the players reach the “end of the road”. At that point, participants assemble and discuss their particular experiences |
| **The Sensitization Program for Geriatric Nurses**  Astill-McNish 1984 [17] | - Game type: role playing - Learning objective: to change the problem behavior of participants - Game objective: to draw the best of different techniques and theories related to aging and package them so that nurses can share them effectively - Players: nurses - Note: the objective of most previous educational programs was changing the attitude of participants instead of the problem behavior | - 5 sessions are held over 1^1/2^ day in small groups of 6-10 with a group leader - During the sessions, players discuss topics related to aging, imagine themselves growing old or losing their precious items, share stories from their past reflecting their real personalities and simulate nursing home patients during eating time - Players exchange ideas, comments and experiences and arrive at conclusions and summaries with the guidance of the group leaders |
| **Geriatrix**  Hoffman 1985 [18] | - Game type: board game with role playing - Learning objective: to prepare students for what they would be learning; to facilitate attitude change about the elderly; to give students a value structure within which they can organize their array of experience - Game objective: to win the most points (represented by chips) in a 90-minute period - Players: 6-8 medical students with experienced clinicians serving as game moderators/judges - Duration: 90 minutes - Note: diverse faculty input is helpful for providing guidance and clinical insights | - The playing board is similar to Monopoly with 24 squares divided into 4 types: fact, concept, crisis and point earning/deducting squares - All players begin with 50 points each. To start, they toss two dices to determine which of the following roles they assume: A physician, a staff member, a family member and an elder - Players then toss dices to move around the board using markers denoting roles. When landing on a square, the players draw a card with a statement and respond accordingly by discussing, acting or earning/loosing points |
| **Geriatric Challenge Bowl**  Crooks 1987 [19] | - Game type: TV game show style based on ‘College Bowl” - Learning objective: to simulate awareness and interest in geriatric patient care among housestaff who did not rotate on the geriatric teaching ward; to intensify the learning experience for housestaff who did - Game objective: to answer the questions correctly in every round - Players: residents in teams each containing: an intern, a junior and senior resident - Duration: 50 minutes - Note: compensations are available at the end for winners | - 8 teams compete in pairs, in 8 rounds: 4 preliminary, 2 semifinal and 1 final - During the rounds, players, as a team, answer questions that vary in formats with each round |
| **An exercise in experiential learning**  Babic 1987 [20] | - Game type: role playing - Learning objective: to increase students’ empathy toward changes that occur with human aging - Game objective: no specific objective identified. - Players: students of an undergraduate class “introduction to gerontology” - Duration: a minimum of 24 hrs - Note: the self selection method for the student experiments and analysis of the breadth are essential part of the design. | - Players select some decrement that is a concomitant of the aging process. Then, they devise a method to simulate the situation for a minimum of 24 hours. After receiving the approval from the instructor, the simulation starts. - After completing the simulation, students fill out an immediate-feedback form, and submit a written report. The players shared their experiences and feelings at the end together. |
| **Aging Game**  McVey 1989 [21]  Related:   - Galanos 1993 [22] - Pacala 1995 (modified version) [23] - Varkey 2006 (modified version) [24] - Pacala 2006 [25] - Henry 2007 (modified version) [26] - Douglass 2008 | *Adapted from “Into Aging” [21]*   - Game type: role playing - Learning objective: to improve empathy and attitude towards elderly; to increase knowledge of geriatrics; to change general beliefs about elders - Game objective: to keep savings, remain independent and retain role identity despite the odds against the player - Players: medical students - Duration: 30 minutes introduction and 90 minutes for role playing - Equipment used: ear plugs, heavy socks, popcorn kernels in the shoes, and taped eyeglasses to simulate disabilities and sensory loss; and paper labels to declare the mental and emotional status | - In phase 1, players choose a name, an old age, a place of residence, and 3 favorite possessions. They keep personal characteristics (self-image chips) and retirement savings (income chips) - In phase II, players interact with a facilitator. They roll dices, toss a wooden ship to land on higher income squares and drawing cards representing life events older people may experience - Players progressively experience loss of savings, independence and self image. They accordingly progress through 3 areas representing respectively independent life style, semi-independent life style, and lifestyle of near total dependence. In parallel, the facilitators change attitude toward them gradually from positive to negative - In phase III, players share experiences and exchange ideas among themselves and the facilitators |
| **Simulation game**  Bachelder 1989 [27] | - Game type: role playing - Learning objective: to improve understanding of sensory changes in the elderly, analyze the functional implications, and facilitate problem-solving approaches for effective adaptation or compensation - Game objective: not mentioned clearly - Players: 1st year occupational therapy students - Note: used Wright’s coping-versus-succumbing theory of attitude change | - Players in pairs proceed through a series of simulated experiences at stations throughout the room - They follow written instructions at each station to simulate sensory changes of elderly: hearing, vision, taste, smell, kinesthesia, and touch - Following each simulated loss the player pair completes a worksheet about functional problems, remaining assets and abilities, personal adaptation and environmental barriers and adaptation to improve or enhance remaining function - Debriefing session at the end to clarify purpose of the activities, to answer questions, and synthesize responses to the experience |
| **A day in life of inpatient**  Cosgray 1990 [28] | - Game type: role playing - Learning objective: to explore helplessness, powerlessness and anger/hostility; to influence in positive way the attitude of staff by using simulation game - Game objective: to simulate the patients and the house staff and to experience different behaviors related to in-patient life - Players: hospital staff - Duration: 45 minutes of role playing and 45 minutes post game discussion | - First, players meet an admission clerk to provide personal information and write precious personal possession on 3 cards that can be put away at anytime for safekeeping - Next, they sit with a psychologist and receive a label on their forehead with descriptive diagnosis sometimes hidden from them - Then, players communicate for 45 min with the nurse, the ward attendant and other staff who apply various negative, positive and detached behaviors - Finally, a 45 min postgame discussion allows brainstorming about “what does this mean to staff?” Participants either players or facilitators share feelings about caretakers and view life from another vantage |
| **Gerontopoly**  Israel 1992 [29] | - Game type: board game - Learning objectives: to understand the demographic, socioeconomic characteristics of the elderly population; to understand the social, physical, psychological, physiological and biological changes associated with aging; to appreciate the impact of the disease, health care system and the environment on the elder’s well being; to develop an empathic understanding of the problems and special needs of the elderly - Game objective: to win the points calculated from a combination of money, functional cards, rehabilitation cards, driver’s license, and chronic disease cards - Players: dental students, hygiene students, sociology students, and faculty development program participants; total number of players: 2-6 - Duration: about one hour | - Each player begins with $15000 in play money, a driver’s license and six function cards. They move counter-clockwise on the board, land on different spaces and react according to the instruction provided in that space. Types of board spaces: Life Event, Doctor Visit, Question Card, and What If Card. Players, themselves are moving on the board - As the game progresses, players explore different feelings about aging, move in or out of assisted living housing, hospital or nursing home, and gain/ loose function’s cards and chronic disease cards |
| **A program to sensitize students to issues of geriatric care**  Turpie 1992 [30] | - Game type: role playing - Learning objective: to sensitize students to the impacts of chronic disease mainly the ones related to aging - Game objective: to experience effects comparable to those produced by some of the disabilities and illnesses of the elderly - Players: medical students - Duration: one and half hour of role playing - Note: facilitators are physicians, occupational therapists or nurses who help introduce the players to issues like feeding, giving instructions and physical impairment | - Players are introduced to the geriatric care through questions followed by short discussions during sessions devoted to diseases biology - Players are then divided to groups of 20, and placed in situations to simulate the disabilities of the elderly - The game is followed by general discussion and evaluation |
| **Old for an evening**  Halloran 1994 [31] | - Game type: role playing - Learning objective: to experience the lifestyle of elderly and to sensitize students to elderly issues - Game objective: to experience the lifestyle of elderly - Players: master’s degree students enrolled in an adult nursing course - Note: volunteers help in simulating the sensory impairment and physical disability of the players; e.g. wrapping the eyes, placing colostomy bags and strapping arms to sides to simulate hemiplegia | - After assigning a tag with new name, age, personality trait and a budget, players proceed to the “finance game” - Players throw checker onto a board, meet with “social worker”, draw cards and experience different aspects of elderly life, physical disability and limitation by following the instructions - After finishing, Players discuss their feelings and ways they will change their practice when nursing elderly patients |
| **You Can Touch This**  Clark 1995 [32] | - Game type: role playing - Learning objectives: to sensitize individuals to the limitations in every day functioning experienced by older people; to help students and professionals understand the physical changes that accompany aging - Game objective: to complete simulation activities - Players: students of introductory psychology, adulthood and aging classes - Equipments used: eyeglasses, walkers and wheelchairs, plastic wrap to simulate vision, hearing and mobility impairment as well as manual dexterity: plywood boards for splints, newspapers, magazines and old medication containers | - A discussion of the physical characteristics of aging and various disabilities precede the simulation exercises - Players experience the multiple aspects of the Simulation Exercises on Aging and Disabilities(SEAD) activities developed by the authors - Post-exercise discussion emphasizes empathy, other affective responses elicited by the activities, potential problems with access encountered in impaired elderly and possible solutions |
| **Resident assessment Instrument** (RAI)  Cipriani 1995 | - Game type: lecture and role playing - Learning objectives: to improve students’ management skills, decision-making capacity, and problem-solving ability and promote positive attitude toward aging - Game objective: to identify residents’ needs, prioritize their problems, write care plans and describe how to implement them, and specify which professionals are needed. - Players: students of health care professions; certified nurses, physical therapist and social workers. - Tools and equipments: audiovisuals, videotapes | - Each course is divide into two sections; - During the first section, students receive instructions about gerontology and geriatric issues through conferences, lectures and classes. - A problem-based learning module follows the first section. 2 interactive methods are used: discussion using case presentation for example and simulation techniques such as role playing. - Students use RAI in all didactic cases to prepare the care plan. RAI consists of minimum data set (MDS) a function-based assessment tool, and resident assessment protocol (RAP) which enables the nurse to identify the patient’s problem and find a care plan |
| **The Geriatric Medication Game**  Oliver 1995 [33]  Evans 2005 | - Game type: role playing - Learning objectives: to increase players’ awareness of the physical, psychological, and financial difficulties which the ambulatory elderly have in handling their medications; to change the attitude towards elderly - Game objective: to finish the tasks required at different stations while retaining characteristics and money - Players: pharmacy students - Duration: less than 1 hour | - In phase I, players choose personal characteristics for themselves as if they were over 65 years old; they also receive information about their disabilities and financial status - In phase II, players rotate over 3-6 tables where they encounter specific tasks and pick adverse reaction cards. They experience medication-related challenges, dehumanizing behaviors, indeterminate fees, and loss of valued characteristics - In phase III, players wrap up and discuss their emotions, the issue of stereotyping, and ways to overcome the barriers encountered |
| **An Aging Simulation**  Lorraine 1998 [34] | - Game type: role playing - Learning objective: to recognize the importance of functional ability in determining the quality of life and patient compliance; to develop proactive clinical approaches to the care of geriatric patients - To identify feelings regarding the experience of functional loss and developing creative problem solving techniques relevant to individual patients - Game objective: to maintain independent activities of daily living (ADL) in spite of a debilitating disease and aids/equipments used - Players: 4th year medical students - Duration: 3 hours - Equipments used: players use glasses, wear foam-filled slippers, tie their ankles and tape their fingers in order to simulate a disability such as vision impairment, neuropathy, narrow-based gait, and arthritis respectively | - 3 hours of simulation take place in a retail store that sells durable medical equipments and supplies, and is divided into three phases: (1) pre-simulation to explain the purpose and the process of all the activities; (2) participation in simulation and; (3) post-simulation discussion facilitated by faculty team to explore the impact of the game and discuss it - After assigning a diagnosis, players use equipments to simulate the real patients they represent and try to accomplish basic ADL or independent ADL (IADL) |
| **As We Grow**  Thomson 1998 [13] | - Game type: role playing - Learning objective: to sensitize nursing assistants in a long term care setting to the aging process and experiences of the elderly - Game objective: discussion only - Players: nursing assistants - Duration: 3^1/2^ hours | - Participants write down 7 of the most important things in their lives (e.g. people, animals, careers, possessions) - A poem detailing the life experience of an elderly person is then read. The participants cross off similar items on their personal list as they are identified in the poem. Participants reflect on their feelings during a debriefing session - The second component of the educational session involves having the participants play ‘‘Into Aging,’’ The game was facilitated by an Overall Game Director and three Table Operators. Following the game, a debriefing session is held |
| **Sexual Dysfunction Trivia Game**  Skinner 2000 [35] | - Game type: board game - Learning objective: to educate staff nurses about sexual dysfunction in the older adult; to dispel the old myths that exists regarding sexual dysfunction in the elderly - Game objective: to collect money - Players: staff nurses; 3-8 players including the Banker - Duration: 1 hour - Note: the game includes a total of 100 trivia questions. Each question has either a multiple-choice format or a true/false format. Questions are based on Erectile dysfunction and impotence related topics | - Board game with 31 fluorescent colored squares around the board’s outer edge - After starting from “Go: collect 100$ for medical expenses” square, the players roll the dice and move forward - When the player lands on a square, he/she responds by following the instructions or answering a trivia question or involuntary waiting until the next turn to roll the die - A Banker is responsible for ensuring the trivia questions are answered correctly and awarding or collecting the money |
| **Senior Safety Solitaire**  Herning 2000 [36]  Related:   - Tumosa 2006 [37] | - Game type: card game - Learning objective: to quantify the effects of teaching games on patient care - Game objective: to identify the safety hazards in pictures of various rooms of a typical house - Players: trainers health related specialties | - The game is based on “ what is wrong with this picture?” protocol - Trainees learn the game during a special session. They get the pictures either by borrowing or buying, and apply them on their clients |
| **Half Full Aging Simulation Experience**  Robinson 2001 [38] | - Game type: role playing - Learning objective: to have students experience aging disabilities, sensitize them to aging problems and change their attitudes towards elderly - Game objective: to experience aging challenges and environmental adaptations - Players: medical students - Equipment used: yellow lenses glasses, cotton balls in the ears, rubber gloves and an immobilizer to simulate the senses and other deficits - Note: this simulation experience is different than previous ones by emphasizing variable adaptation methods that can have big impact on quality of life | - Players start with a short presentation by a geriatrician about the decrements and changes related to aging while they learn to focus on the strengths that older individuals retain symbolized by “half full” cup - During the simulation game, players experience the deficits of senses and the difficulty in performing various common tasks. They also experience how simple environmental adaptations can change the function and the quality of life - A discussion session follows the game where players learn about variety of adaptation methods |
| **Who wants to be a hundredaire?**  McCahan 2002 [39] | - Game type: TV game show style - Learning objective: to provide continuing education for certified nursing assistants; to increase their knowledge about frail old adults who have intense care needs like nutritional status and end of life-care; to create an interdisciplinary approach in learning that involves all staff - Game objective: to answer questions correctly - Players: certified nursing assistants - Duration: 50 minutes for the game and 10 minutes for discussion - Rewards: cash, gift certificates, service awards or other - Note: representatives from each discipline write questions related to their specialty | - Follows the rules of the TV game show “Who Wants to Be a Millionaire” with questions and answers in an entertaining way - As questions increase in difficulty rewards increase. A player may opt out at any time and win the amount in hand - The player has the same lifelines of the TV show such as “Ask the audience” “50-50” and” ask a friend” - Game is followed by 10 minutes of group discussion |
| **A Simulation About Functional Change and Aging**  Wood 2002 [40] | - Game type: role playing - Learning objective: to develop an appreciation of the effects age-related changes in sensory-motor function have on daily living activities - Game objective: to live the experience of an elderly with sensory deficits and mobility difficulties - Players: undergraduate students - Equipment used: tools and equipments to simulate the impairments and receive safety instructions before leaving | - Small groups of 3-4 players are assigned either a caregiver or a patient role - Patients encounter sensory deficits as well as mobility impairments. They leave the classroom accompanied by their caregivers to experience the new functional status while attending to regular daily activities for about 30 min - After the role playing, students discuss their experiences with physiological aging |
| **Introduction to aging**  Altpeter 2003 [30, 41] | - Game type: Q/A followed by debriefing session IS IT A GAME? - Learning objective: to uncover students’ attitudes about their own aging using Life Expectancy Calculator Exercises; to sensitize students to the aging experience, the diversity of the aging population, and the value of intergenerational communication - Game objective: discussion only - Players: undergraduate students - Equipment used: “Living to 100 Life expectancy Calculator”, and “The Longevity Game”, Data processing software | - After the introduction to the game objectives, a brief lecture demonstrates the rapidly growing elderly population - Players meet each other through a few brief ice breaker small group discussion exercises - Discussion moves from abstract ideas about youth, generational differences to more deeply personalized experience using the Life Expectancy Calculator Exercises - Players calculate their expected age and answer questions about their reaction to the aging process; how long they may live, health and income applications, preventive steps and the influences on retirement and career - A simple content analysis using computer software follows the game to analyze meanings and helps to code the attitudinal responses and organize the students’ written responses |
| **The Age Game**  Kennedy 2004 [42] | - Game type: board game - Learning objectives: to integrate the pharmaceutical care issues that are emphasized in the *Geriatric Medication Game* [33] and the social issues emphasized in *SEAD activities* [32]; to recognize the limitations and barriers that the senior population faces; to expose players to practical issues encountered while caring for the elderly; to develop participants’ critical thinking skills about their role in caring and to prepare them to counsel the older adult - Game objective: to reach the last board’s space - Players: pharmacy students - Duration: 2 hours - Note: a guide that gives the correct answers or information for the scenario cards is provided for the instructor. A facilitator rotates among the groups | - Players are divided in groups of 3-5 - Each player rolls a dice and advances the marker “game piece” accordingly on the identified spaces - When a player lands on a space with instruction for movement, he/she simply follows it - When a player lands on a solid-color space (without instructions), he/she takes a scenario card from the center of the game board, reads it and follows the instructions. The player may advance or retreat based on his/her performance - Possible scenarios: submit to a cognitive assessment (Mini-Mental Status Exam) and move backward one space for every missing point, simulate decreased vision and attempt to read, or simulate hemiparesis and attempt to wear a shirt and button it before a fellow player can count to 30. Other scenario cards include explanations of a geriatric-related topic like urine incontinence and dysphagia - Players can not advance any further once they land on a solid-color space twice during their turn |
| Schuldberg 2005 | - Game type: role playing - Learning objective: to develop an appreciation of the effects of age-related visual impairment on daily living activities - Game objective: to live the experience of an elderly with different types of visual impairment. - Players: social work students - Equipment used: tools and equipments to simulate the impairments | - Class was divided into groups. - Players in every group rotate the use of the glasses that simulate the visual impairment and write their observation and feelings on a “response sheet” - The students are encouraged to view themselves as researchers, writing field notes that detailed their experiences - The response sheet included also questions regarding their view of elderly and the changes of this view after the game, as well as the learning experience from this exercise - After the exercise, students receive a lecture on the etiology and symptoms of the impairments where students share their feedback from the response sheet - At the end, students provide suggestions and feedback |
| **Future-self worksheet**  Remnet (1989)  Masters (2006) | - Game type: mental exercise - Learning objective: to educate students about aging process and how they can influence their own senescence. - Game objective: players are challenged to visualize their own aging experience and to create graphic representation of their visualization. - Players: students enrolled in introductory course about gerontology. - No special equipments are used. | - Players undergo a process of guided thinking about the future and what it will be like to belong to a different age cohort. - Participants complete squares with drawings and words that describe themselves when they are elderly. Squares represent areas of life such as physical, environmental, social activities, financial support and psychological/spiritual self. - Players are allowed to use internet clip-art, family photographs, and pictures from magazines. - Following the exercise, the instructors use the information from the squares to customize lecture materials to student’s interests and concerns. |

**References**

1. Chaisson G: **Life-cycle: Simulating the problems of aging and the aged.** *Health Education Monographs* 1977, **5:**28-35.

2. Hoffman TL, Reif SD: *Into Aging : A Simulation Game.* Thorofare, NJ: Charles B. Slack; 1978.

3. Bonstelle SJ, Govoni AL: **Into aging: exploring aging through games.** *Rehabilitation Nursing* 1984, **9:**23.

4. Nolan DM: **The analysis of a simulation technique for developing attitude change towrad elderly among health care providers.** Boston University, 1985.

5. Hoyt JM: **An investigation of a simulation game and the effects of gaming on nurses aides' attitudes toward the elderly in long term care.** *A dissertion presented to the faculty of the school of education counseling and educational psychology program* 1987.

6. Wirth CA: **The relationship between participation in an aging simulation game and the attitudes of nursing students toward the elderly.** 1987.

7. Marte AL: **How does it feel to be old? Simulation game provides "into aging" experience.** *Journal of Continuing Education in Nursing* 1988, **19:**166-168.

8. Jadlowksi SL: **The effect of participation in an aging simulation game on the attitudes of senior associate degree nursing students toward the elderly.** 1991.

9. Hahn-Marsh CV: **Effect of the simulation game "into aging" on nurses' attitudes toward the elderly.** 1992.

10. Seibert B: **Effects of a simulation game on nursing home nurses' aides' attitudes toward elderly individuals.** *M.S.* University of Missouri - Columbia, 1992.

11. Leblanc PA: **Attitudes of nursing students toward the elderly as influenced by lecture-discussion with and without simulation.** *PH.D. 91 p.* The University of Southern Mississipi, 1995.

12. Khazadian-Figueroa MR, Johnson E: **Simulation game: a tool for staff development and its effects on staff behavioral outcomes.** *Journal of Nursing Staff Development* 1997, **13:**223-226.

13. Thomson M: **A Nursing Assistant Training Program in a Long Term Care Setting.** *Gerontology & Geriatrics Education* 1998, **19:**23 - 35.

14. Robertson D, Brocklehurst J: **The aging game: a new teaching method in geriatric medicine.** *Journal of the American Geriatrics Society* 1981, **29:**576-578.

15. Lye MD: **The ageing game.** *Age & Ageing* 1983, **Suppl:**36-38.

16. Menks F: **The use of a board game to simulate the experiences of old age.** *Gerontologist* 1983, **23:**565-568.

17. Astill-McNish S: **A sensitization program for geriatric nurses: games that make you care.** *Canadian Nurse* 1984, **80:**19-24.

18. Hoffman SB, Brand FR, Beatty PG, Hamill LA: **Geriatrix: A role-playing game.** *Gerontologist* 1985, **25:**568-572.

19. Crooks V, Yoshikawa TT: **Geriatric Challenge Bowl: an innovative teaching approach.** *Gerontology & Geriatrics Education* 1987, **7:**67-79.

20. Babic AL, Crangle ML: **Simulation Techniques for Education in Gerontology: An Exercise in Experiential Learning.** *Educational Gerontology*

1987, **13:**183-191.

21. McVey LJ, Davis DE, Cohen HJ: **The 'aging game'. An approach to education in geriatrics.** *JAMA* 1989, **262:**1507-1509.

22. Galanos AN, Cohen HJ, Jackson TW: **Medical education in geriatrics: The lasting impact of the aging game.** *Educational Gerontology* 1993, **19:**675-682.

23. Pacala JT, Boult C, Bland C, O'Brien J: **Aging game improves medical students' attitudes toward caring for elders.** *Gerontology & Geriatrics Education* 1995, **15:**45-57.

24. Varkey P, Chutka DS, Lesnick TG: **The Aging Game: improving medical students' attitudes toward caring for the elderly.** *Journal of the American Medical Directors Association* 2006, **7:**224-229.

25. Pacala JT, Boult C, Hepburn K: **Ten years' experience conducting the Aging Game workshop: was it worth it?** *Journal of the American Geriatrics Society* 2006, **54:**144-149.

26. Henry BW: **Effects of participation in an aging game simulation activity on the attitudes of Allied Health students toward older adults.** *The internet journal of allied health sciences and practice* 2007, **5**.

27. Bachelder J: **Effectiveness of a simulation activity to promote positive. attitudes and perceptions of the elderly.** *Educational Gerontology* 1989, **15:**363- 375.

28. Cosgray RE, Davidhizar RE, Grostefon JD, Powell M, et al.: **A Day in the Life of an Inpatient: An experiential game to promote empathy for individuals in a psychiatric hospital.** *Archives of Psychiatric Nursing* 1990, **4:**354-359.

29. Israel MD, Dolan TA, Caranasos GJ: **Gerontopoly: Development and testing of a new game in geriatric education.** *Gerontology & Geriatrics Education* 1992, **12:**17-30.

30. Turpie ID, Bloch R, Edwards M, Rangachari P, Patterson CJ, Tainsh SM: **A program to sensitize students to issues of geriatric care.** *Academic Medicine* 1992, **67:**304-306.

31. Halloran L, Dean L: **Old for an evening: an experiential learning game.** *Journal of Nursing Education* 1994, **33:**155-156.

32. Clark MC, Foos PW, Faucher MH: **You can touch this: Simulation exercises for aging and disability.** *Educational Gerontology* 1995, **21:**643 - 651.

33. Oliver CH, Hard PD, Beavers M, Gibbs E, B. G, Miller K: **Experiential Learning About the Elderly: The Geriatric Medication Game** *American Journal of Pharmaceutical Education* 1995, **59:**155-158.

34. Lorraine V, Allen S, Lockett A, Rutledge CM: **Sensitizing students to functional limitations in the elderly: an aging simulation.** *Family Medicine* 1998, **30:**15-18.

35. Skinner KD: **Creating a game for sexuality and aging: the Sexual Dysfunction Trivia game.** *Journal of Continuing Education in Nursing* 2000, **31:**185-189.

36. Herning M: **Senior Safety Solitaire.** In *PT-Magazine of Physical Therapy*, vol. 8. pp. 43-47; 2000:43-47.

37. Tumosa N, Morley JE: **The use of games to improve patient outcomes.** *Gerontology & Geriatrics Education* 2006, **26:**37-45.

38. Robinson SB, Rosher RB: **Effect of the "half-full aging simulation experience" on medical students' attitudes.** *Gerontology & Geriatrics Education* 2001, **21:**3-12.

39. McCahan C: **Improving CNA education with a game show.** *Geriatric Nursing* 2002, **23:**200-202.

40. Wood: **Experimental learning for undergraduates. A simulation about functional change and ageing.** *Gerontol Geriatr Education* 2002

37-48.

41. Altpeter M, Marshall VW: **Making Aging "Real" for Undergraduates.** *Educational Gerontology* 2003, **29:**739 - 756.

42. Kennedy DH, Fanning KD, Thornton PL: **The Age Game: An Interactive Tool to Supplement Course Material in a Geriatrics Elective.** *American Journal of Pharmaceutical Education* 2004, **68:**1-6.
