# Supplementary material for: Educational games in geriatric medicine education: a systematic review
Source: BMC Geriatr. 2010 Apr 23;10:19. doi: 10.1186/1471-2318-10-19 (PMC2867807; doi:10.1186/1471-2318-10-19)
Supplement: Additional file 4 — Characteristics of randomized controlled trials included in the systematic review. Characteristics of randomized controlled trials included in the systematic review. [file 1471-2318-10-19-S4.DOC]

Characteristics of randomized controlled trials included in the systematic review

| **Study** | **Study design** | **Intervention** | **Participants** | **Outcomes** | **Methodological quality** | **Results** |
| --- | --- | --- | --- | --- | --- | --- |
| Chaisson 1977 [1] | - Randomized control trial - Pretest and posttest evaluation (immediately post intervention) | - *“Life Cycle”* - Intervention:   Field experiment in a nursing home, divided into 3 sessions for a total of 10 hrs including the introduction.   - Control: no intervention | - 40 healthcare professionals in a nursing home (no number reported for intervention & control) - Country: USA | - Attitude toward the elderly: Kogan Attitude toward Old People scale [2] - Satisfaction | - Allocation concealment: not reported. - Comparison of baseline characteristics: not reported - Baseline test: yes - Protection against contamination: not reported - Standardized outcome measurement tool: yes - Analytic approach described: not clearly - Follow up: 100% answered questionnaire | Attitude toward the elderly:   - No statistically significant changes in attitudes from pre to post in either group - No statistically significant difference between the 2 groups   Satisfaction:   - 100% of participants reported the training was a pleasant and teaching experience and that they recommended it to others |
| Bachelder 1989 [3] | - Controlled trial - Pretest and posttest evaluation (1 week post intervention) | - *“Simulation using Wright’s coping-Vs. succumbing theory of attitude change”* - Intervention: simulation activity in a lab session concurrent with lecture presentations - Control: free time to work on unfinished projects | - 44 first year students in occupational therapy ((intervention: 22; control: 22) - Country: USA | - Attitude toward the elderly:   - Attitude toward the Elderly scale (AE) scale [3]   - Perception of the elderly as potential recipients of services: Attitude toward Practice Areas (APA) [3] | - Comparison of baseline characteristics: no - Baseline test: yes - Protection against contamination: not reported - Standardized outcome measurement tool: yes - Analytic approach described: yes   Follow up: not reported | Attitude toward the elderly:   - AE: Positive change (+0.73) in the intervention and negative change (-1.54) in control group; difference between 2 groups for change not statistically significant - APA, part I: no significant differences between 2 groups for change. - APA, part II: statistical significance of differences between 2 groups for change not reported |
| Nolan 1985 [4] | - Randomized controlled trial - Posttest evaluation (immediately and 3 week post intervention) | - *“Into aging”* - Intervention group: playing the game - Control: no intervention | - 65 nursing staff in 2 long term care institutions (number per group not reported) - Country: USA | - Attitude toward the elderly: Kogan Attitude toward Old People Scale [2] | - Allocation concealment: not reported - Comparison of baseline characteristics: not reported - Baseline test: yes - Protection against contamination: not reported - Standardized outcome measurement tool: yes - Analytic approach described: not clearly - Follow up: not reported | Attitude toward the elderly:   - No statistically significant difference between the 2 groups or between the immediate and 3 weeks posttest scores of the intervention group. |
| Hoyt 1987 [5] | - Randomized control trial - Pretest and posttest evaluation (3 weeks post intervention) | “Into Aging”   - Intervention: one session of the game - Control: no intervention | - 26 Nursing aides of long-term care (number per group not reported) - Country: USA | - Attitude toward the elderly: Kogan Attitude toward Old People scale [2] | - Allocation concealment: not reported - Comparison of baseline characteristics: no - Baseline test: yes - Protection against contamination: not reported - Standardized outcome measurement tool: yes - Analytic approach described: yes - Follow up: not reported | Attitude toward the elderly:   - No statistically significant changes in attitudes from pre to post in either group - No statistically significant difference between the 2 groups in terms of change from pre to post |
| Seibert 1992 [6] | - Randomized control trial - Pretest and posttest evaluation (immediately post and 3 weeks post intervention) | - *“Into Aging”* - Intervention: playing the game for 45 minutes followed by debriefing period - Control: no intervention | - 16 nurses aides at a skilled care nursing home (of 28 potential participants) (intervention: 8; control: 8) - Country: USA | - Attitude toward the elderly: Kogan Attitude toward Old People scale [2] | - Allocation concealment: no - Comparison of baseline characteristics: not balanced - Baseline test: yes - Protection against contamination: not reported - Standardized outcome measurement tool: yes - Analytic approach described: yes - Follow up: 100% | Attitude toward the elderly:   - No statistically significant difference between the 2 groups in terms of change from pre to immediately post or 3 weeks post |
| LeBlanc 1995 [7] | - Randomized control trial - Pretest and posttest evaluation (immediately post, and 8 weeks post intervention) | - *“Into Aging”* - Intervention: playing the game during 2-hour session - Control: lecture-discussion about attitude toward aging | - 98 associate degree nursing program students (intervention: 48; control: 50) - Country: USA | - Attitude toward the elderly: Kogan Attitude toward Old People scale [2]. | - Allocation concealment: no - Comparison of baseline characteristics: not reported - Baseline test: yes - Protection against contamination: not reported - Standardized outcome measurement tool: yes - Analytic approach described: not clearly - Follow up: not reported | Attitude toward the elderly:   - No statistically significant difference between the 2 groups in terms of change from pre to immediately post - Change from pre to 8 weeks post was statistically higher in the intervention group - The mean and SD of scores for the intervention and control group were: pretest: 148.48 (12.47); 148.82 (13.04); immediate post test 157.69 (12.00); 158.98 (13.34); 8 weeks post test: 161.94 (12.95); 157.56 (12.87) |
| Pacala 1995 [8] | - Controlled trial (those choosing to participate were included in intervention group) - Pretest and posttest evaluation (immediately post) | - *“Aging Game”* - Intervention: 3-hour elective *Aging Game* workshop - Control: no intervention | - 55 fourth year medical students during ambulatory medicine course (intervention:39, control: 16) - Country: USA | - Attitude towards the elderly   - Modified Maxwell Sullivan Attitude Scale [9]   - Aging Semantic Differential scale (ASD) [10] - Knowledge of geriatrics: self developed tool - Satisfaction | - Comparison of baseline characteristics: not balanced - Baseline test: yes - Protection against contamination: not reported - Standardized outcome measurement tool: yes - Analytic approach described: yes - Follow up: 100% | Attitude toward the elderly:   - Change from pre to post were significantly higher in intervention: 1.63 vs. -1.07 (SD not reported) p =0.009 - No statistically significant difference between the 2 groups for beliefs, empathy, or knowledge   Satisfaction   - Ratings of the workshop were overall positive |
| Oliver 1995 [11] | - Controlled trial - Pretest and posttest evaluation (at the beginning of the semester and post intervention) | *“The Geriatric Medication Game”*   - Intervention: one hour of role playing during professional communication class | - 63 pharmacy students (intervention:48, control: 15) - Country: USA | - Attitude toward elderly: 9 semantic differential questions (6-point Likert scale) | - Comparison of baseline characteristics: no - Baseline test: yes - Protection against contamination: no - Standardized outcome measurement tool: no - Analytic approach described: not clearly - Follow up: 100% completed post-test evaluation | Attitude toward the elderly:   - The posttest score was significantly different from pretest no game, pretest game, and posttest game scores (p<0.05) |

**References**

1. Chaisson, G., *Life-cycle: Simulating the problems of aging and the aged.* Health Education Monographs, 1977. **5**(Suppl 1): p. 28-35.

2. Kogan, N. and N. Kogan, *Attitudes toward old people: the development of a scale and an examination of correlates.* Journal of Abnormal & Social Psychology, 1961. **62**: p. 44-54.

3. Bachelder, J., *Effectiveness of a simulation activity to promote positive. attitudes and perceptions of the elderly.* Educational Gerontology, 1989. **15**(4): p. 363- 375.

4. Nolan, D.M., *The analysis of a simulation technique for developing attitude change towrad elderly among health care providers*. 1985, Boston University: Boston p. 111 pages.

5. Hoyt, J.M., *An investigation of a simulation game and the effects of gaming on nurses aides' attitudes toward the elderly in long term care.* A dissertion presented to the faculty of the school of education counseling and educational psychology program, 1987.

6. Seibert, B., *Effects of a simulation game on nursing home nurses' aides' attitudes toward elderly individuals*. 1992, University of Missouri - Columbia: United States -- Missouri.

7. Leblanc, P.A., *Attitudes of nursing students toward the elderly as influenced by lecture-discussion with and without simulation*. 1995, The University of Southern Mississipi.

8. Pacala, J.T., et al., *Aging game improves medical students' attitudes toward caring for elders.* Gerontology & Geriatrics Education, 1995. **15**(4): p. 45-57.

9. Maxwell, A.J. and N. Sullivan, *Attitudes toward the geriatric patient among family practice residents.* J Am Geriatr Soc, 1980. **28**: p. 341-45.

10. Rosencranz, H.A. and T.E. McNevin, *Aging semantic differential*, in *Research Instruments in Social Gerontology*, D.J. Mangen and W.A. Peterson, Editors. 1982, University of Minnesota: Minneapolis.

11. Oliver, C.H., et al., *Experiential Learning About the Elderly: The Geriatric Medication Game* American Journal of Pharmaceutical Education 1995. **59**(2): p. 155-8.
