# Supplementary material for: Educational games in geriatric medicine education: a systematic review
Source: BMC Geriatr. 2010 Apr 23;10:19. doi: 10.1186/1471-2318-10-19 (PMC2867807; doi:10.1186/1471-2318-10-19)
Supplement: Additional file 5 — Validated tools for assessing attitude outcome. Validated tools for assessing attitude outcome. [file 1471-2318-10-19-S5.DOC]

**Validated tools for assessing attitude outcome**

- **Aging Semantic Differential scale (ASD)** [1]: measures attitudes towards the elderly. It directs respondents to indicate the extent to which 32 polar adjectives (e.g., exciting/dull, progressive/ old-fashioned, busy-idle, independent/ dependent) best describe 35-year-old and then 70-year-old adults. Lower values indicate more positive view. The following four subscales are used: the instrumental-ineffective dimension, autonomous-dependent, personal acceptability-unacceptability and integrity.
- **Maxwell Sullivan Attitude scale** [2]: measures respondents’ general attitudes toward older patients (such as “I will welcome elderly patients into my practice”) as well as respondents’ views regarding the time and energy older patients require and their therapeutic potential (e.g., “Elderly patients take medicines as prescribed”). Students report whether they definitely agree (“1”) to definitely disagree (“5”) with each statement.
- **Kogan Attitude Toward Old Persons (ATOP) scale** [3]: is a one-dimensional measurement tool so that an attitude index is computed as an overall mean of all 34 items. Each item’s value ranges between 1 (disagree strongly) to 7 (agree strongly) with higher scores indicating more positive attitudes.
- **Attitude toward the Elderly (AE) measure** [4]: consists of 55 items representing 3 research content areas: characteristics of old people, the service needs of old people and working with older people which includes three level of effects: a) awareness, b) valuing and c) willingness to respond. Each item is rated using a (Likert) scale along a 4-point continuum of agreement (with no neutral point)
- **Attitude toward Practice Areas (APA)** [4]: a questionnaire constructed to measure subjects’ perceptions of the elderly as potential recipients of their services. It includes 2 parts ; the first is (7) “yes-no” questions while the second consists of (8) “rank-order” questions of seven populations regarding their desirability as patients, suitability of occupational therapy for their problem and the benefit of specific occupational therapy for their problems.

**References**

1. Rosencranz HA, McNevin TE: **Aging semantic differential.** In *Research Instruments in Social Gerontology.* Edited by Mangen DJ, Peterson WA. Minneapolis: University of Minnesota; 1982

2. Maxwell AJ, Sullivan N: **Attitudes toward the geriatric patient among family practice residents.** *J Am Geriatr Soc* 1980, **28:**341-345.

3. Kogan N, Kogan N: **Attitudes toward old people: the development of a scale and an examination of correlates.** *Journal of Abnormal & Social Psychology* 1961, **62:**44-54.

4. Bachelder J: **Effectiveness of a simulation activity to promote positive. attitudes and perceptions of the elderly.** *Educational Gerontology* 1989, **15:**363- 375.
